# Urinary bladder matrix for lower extremity split-thickness skin graft donor site

**DOI:** 10.1093/jscr/rjad529

**Published:** 2023-09-24

**Authors:** Sydney Bormann, Zachary Lawrence, Heather Karu

**Affiliations:** University of South Dakota Sanford School of Medicine, 1400 W 22nd St, Sioux Falls, SD 57105, United States; Department of Surgery, University of South Dakota Sanford School of Medicine, 1400 W 22nd St, Sioux Falls, SD 57105, United States; Department of Plastic and Reconstructive Surgery, Sanford Health, 1500 W 22nd St, Sioux Falls, SD 57105, United States

**Keywords:** split-thickness skin graft donor site, dermal substitute, urinary bladder matrix

## Abstract

Split-thickness skin grafts (STSG) are commonly used to treat soft-tissue defects. Harvesting a STSG creates an additional partial thickness wound at the donor site which must be managed. Many dressings are commercially available for the management of STSG donor sites; however, there is no evidence-based consensus on optimal dressing for site management. Urinary bladder matrix (UBM) is an extracellular matrix that acts as a structural support for tissue remodeling and provides molecular components for repair. Common clinical applications of UBM include coverage of deep wounds, burns, and irradiated skin. Skin grafting from the lower extremities poses a challenge due to the increased dermal tension. UBM-based reconstruction is an alternative method of managing lower extremity skin graft donor sites. This case study demonstrates the use of UBM in the reconstruction of a STSG donor site of the anterolateral thigh, which resulted in satisfactory healing, no pain, and excellent cosmetic and functional outcomes.

## Introduction

Large soft-tissue defects are traditionally treated with immediate full- or partial-thickness skin grafts [1]. Split-thickness skin grafting (STSG), the process of separating a section of epidermis and dermis from the donor site and transplanting it to the recipient site, is a commonly used reconstructive technique. Harvesting a STSG creates an additional partial thickness wound at the donor site which must be managed [2]. Donor site management is aimed at preventing morbidity, including pain, bleeding, infection, scarring, decreased sensation, delayed healing, and poor cosmesis [1]. Consequently, many products, such as mesh gauze or films, are traditionally used to cover the skin graft donor sites with hopes to improve wound outcomes. Hyalomatrix (Fidia Advanced Biopolymers, Padua, Italy), a bilayered bioresorbable dermal substitute, has recently been shown to minimize morbidity and improve donor site healing [1]. This case study demonstrates the use of urinary bladder matrix (UBM) (Acell Lafayette, IN), a different type of dermal substitute, in lower extremity STSG donor site reconstruction.

## Case presentation

A 35-year-old male diagnosed with necrotizing fasciitis of the right lower extremity was transferred to our facility from a rural hospital to undergo irrigation and debridement with intraoperative placement of a subcutaneous wound VAC. Plastic surgery was consulted for skin grafting after serial debridements were complete. The wound, which measured 4.4 × 14.1 cm and extended to the level of the soleus muscle, was closed 3 days later with a 0.014-inch STSG harvested from the anterolateral left thigh in one continuous piece. Epinephrine-soaked gauzed was applied to the donor site to aid with hemostasis and then the graft was anchored to the wound edges circumferentially with interrupted 4-0 gut sutures. The graft was covered with a layer of nonadhering dressing, and a wound VAC was placed.

**Figure 1:**
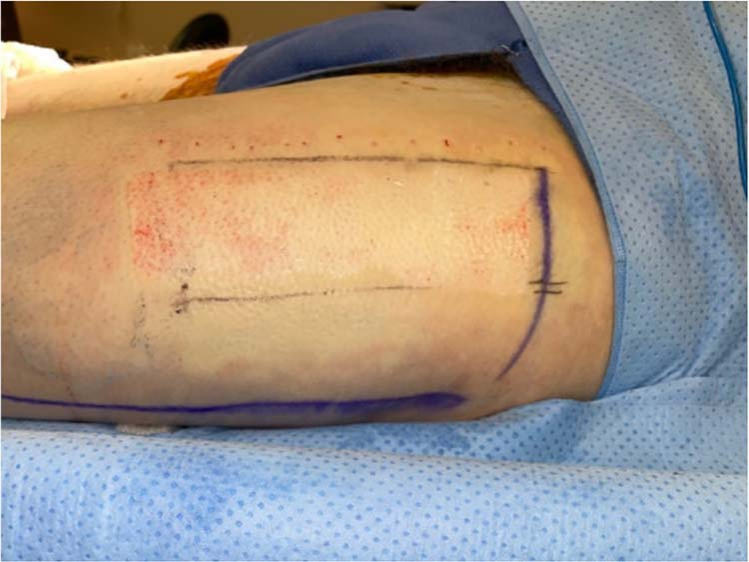
Intraoperative donor site following skin graft harvesting.

**Figure 2:**
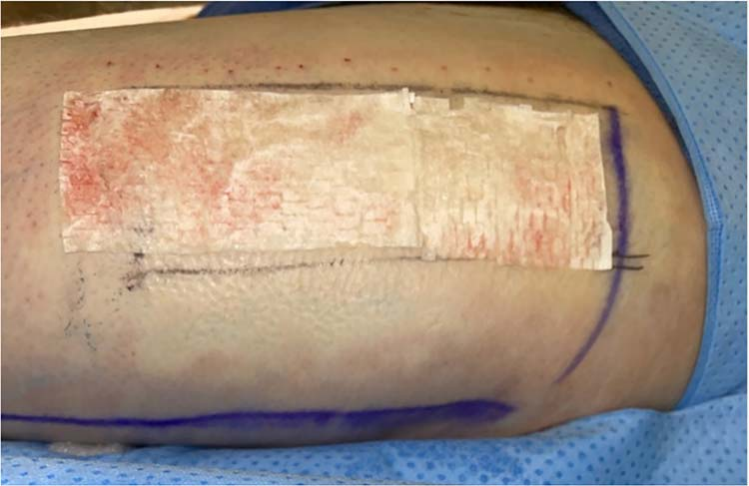
Intraoperative donor site covered with 7 × 10 cm UBM (Acell Lafayette).

**Figure 3:**
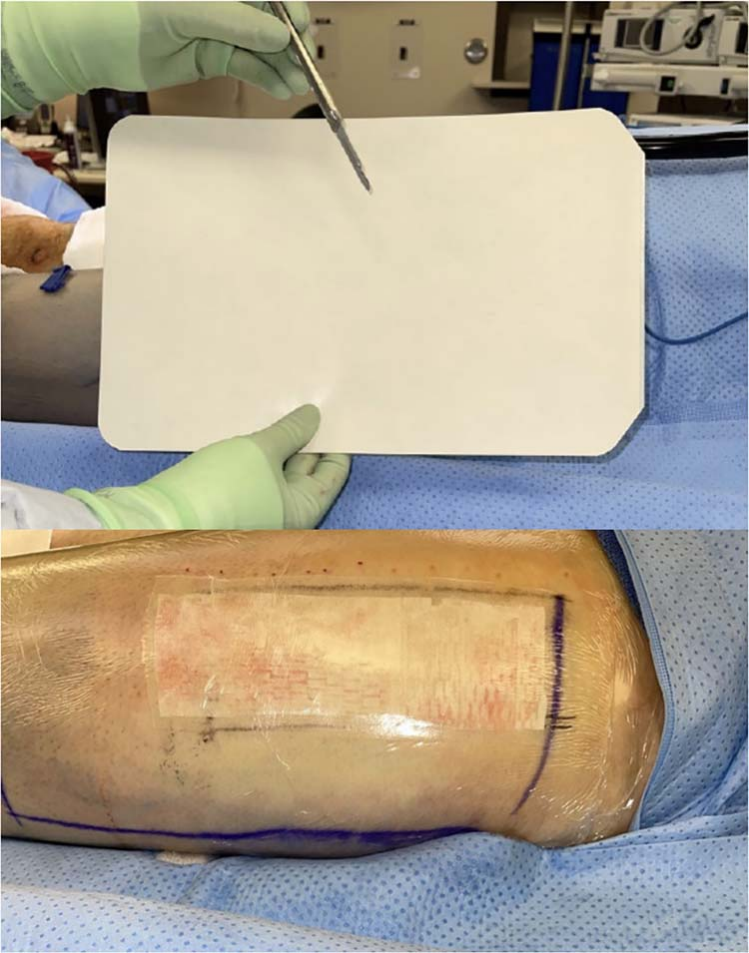
Intraoperative donor site covered with perforated Tegaderm™.

**Figure 4:**
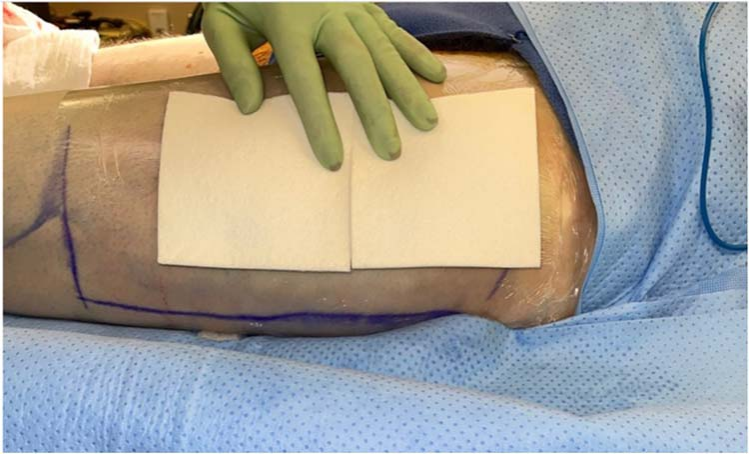
Intraoperative donor site covered with Drawtex®.

**Figure 5:**
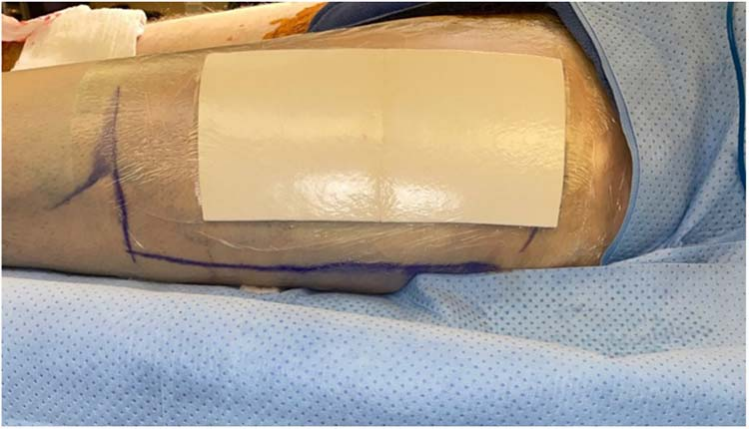
Intraoperative skin graft donor site with dressings in place.

The left anterolateral thigh donor site (Fig. 1) was covered with a single 7 × 10 cm sheet of UBM (Acell Lafayette) (Fig. 2). A Tegaderm™ containing many small perforations created with a surgical scalpel was placed over the UBM, followed by Drawtex®, to wick away excess fluid. All components were then covered with a large intact Tegaderm™ and the dressing remained in place for 1 week (Figs 3–5).

**Figure 6:**
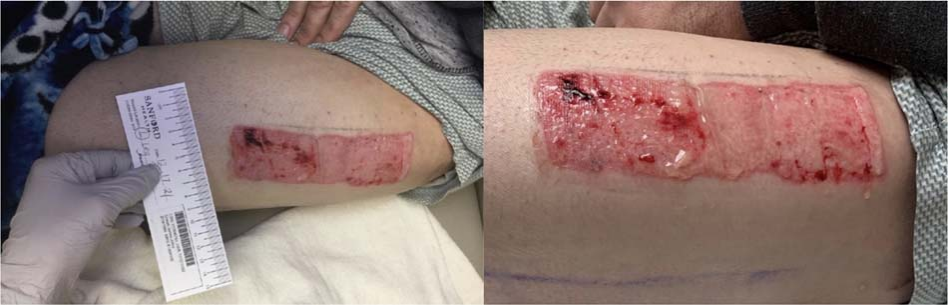
Healing donor site postoperative day 7.

**Figure 7:**
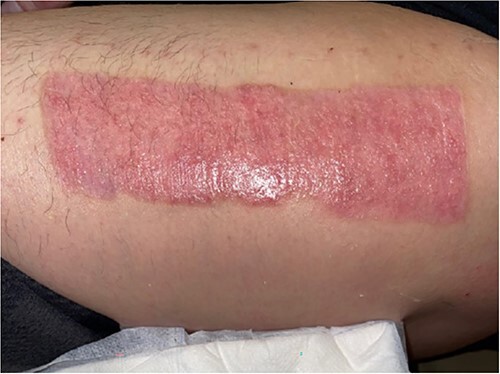
One-month postoperative healing donor site.

**Figure 8:**
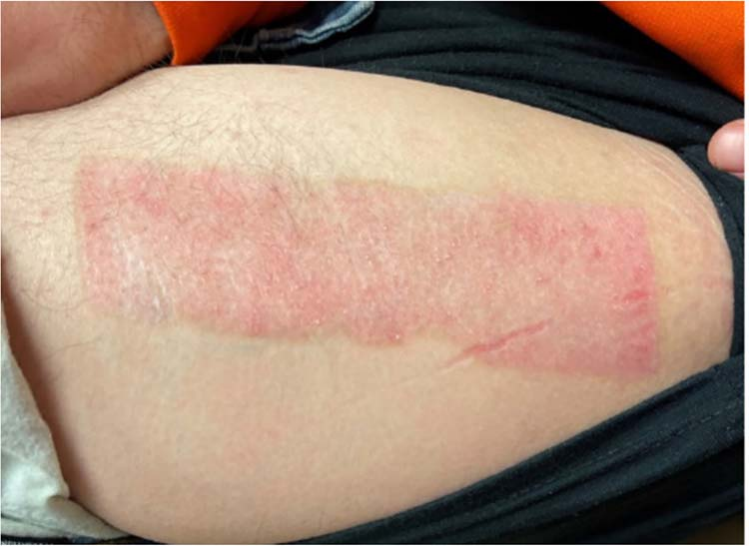
Two-month postoperative healed donor site.

The donor site dressing was removed 1 week postoperatively, revealing a well-healing wound with no infectious signs or symptoms (Fig. 6). The patient reported no pain at the site. The patient was advised to apply Xeroform® and gauze pads to the donor site twice daily. One month postoperatively, the left thigh donor site demonstrated excellent healing with epithelized pale pink tissues (Fig. 7). The patient was advised to continue to apply moisturizing ointment and dressings to the donor site. Two months postoperatively, the left thigh donor site and right lower extremity skin graft site were well healed without complication (Fig. 8).

## Discussion

STSG involves excising the epidermis and part of the dermis to form a graft used for the coverage of a tissue defect caused by trauma, burns, surgical resection, and acute and chronic wounds. The dermis that remains facilitates healing of the donor site and reepithelialization typically occurs within 7–14 days [3]. Proper management of STSG donor sites is critical to promoting healing while maximizing patient comfort and skin cosmesis [2]. Many dressings are commercially available for the management of STSG donor sites; however, there is no evidence-based consensus on optimal dressing for site management [4]. In the absence of consensus on the best practice for STSG donor site management, additional products and techniques have been developed in an attempt to facilitate donor site healing.

Extracellular matrices (ECM) are naturally derived products with clinical applications, including tissue reconstruction and wound management. ECM is derived from many biologic tissues, including heart valves, blood vessels, skin, nerves, skeletal muscle, tendons, ligaments, small intestine, liver, and urinary bladder [5]. UBM acts as a structural support for tissue remodeling and provides molecular components for repair [6]. UBM is demonstrated to be one of the few ECM products that contain an intact basement membrane which supports and facilitates the growth of epithelial cells [7]. It also contains antimicrobial properties which may help facilitate wound healing and tissue regeneration [8]. UBM grafts reduce pain, facilitate epithelial remodeling, and accelerate rates of wound healing [9].

Common clinical applications of UBM include coverage of deep wounds, burns, and irradiated skin. Porcine UBM has been shown to be an effective alternative to flap coverage in patients with orthopedic wounds that have exposed tendon and bone [6]. In traumatic combat-related wounds, UBM facilitates soft-tissue reconstruction by establishing a neovascularized soft-tissue base [10]. UBM also significantly accelerates the healing of irradiated wounds and has shown promise as a therapy for deep partial-thickness extremity burns [9, 11].

Although current literature supports UBM as an alternative to the traditional grafting of fasciocutaneous free flap donor sites [12], there is limited data on the use of UBM for STSG skin graft donor sites. One article highlights satisfactory results when UBM was applied to four full-thickness skin graft donor sites in the preauricular area [13]. Skin grafting from the lower extremities poses a challenge due to the increased dermal tension caused by limited tissue availability. Although existing literature has demonstrated the effectiveness of UBM in treating lower extremity wounds and burns, there is limited research assessing the outcomes of the reconstruction of lower extremity skin graft donor sites using UBM. Our study is one of the few cases demonstrating pain-free healing and satisfactory cosmetic and functional results of a UBM-covered STSG donor site, and it is the only known study demonstrating the use of UBM for a STSG donor site on the lower extremity. Further studies are needed to determine the place of UBM in STSG donor site reconstruction.

## Conclusion

This case study demonstrates that the use of UBM in the management of a STSG donor site results in excellent cosmetic and functional outcomes.
